# Cause or consequence? The role of IL-1 family cytokines and receptors in neuroinflammatory and neurodegenerative diseases

**DOI:** 10.3389/fimmu.2023.1128190

**Published:** 2023-05-08

**Authors:** Diana Boraschi, Paola Italiani, Paola Migliorini, Paola Bossù

**Affiliations:** ^1^ Shenzhen Institute of Advanced Technology (SIAT), Chinese Academy of Sciences (CAS), Shenzhen, China; ^2^ Institute of Biochemistry and Cell Biology (IBBC), National Research Council (CNR), Naples, Italy; ^3^ Stazione Zoologica Anton Dohrn (SZN), Napoli, Italy; ^4^ China-Italy Joint Laboratory of Pharmacobiotechnology for Medical Immunomodulation, Shenzhen, China; ^5^ Clinical Immunology and Allergy Unit, Department of Clinical and Experimental Medicine, University of Pisa, Pisa, Italy; ^6^ Laboratory of Experimental Neuro-psychobiology, Department of Clinical and Behavioral Neurology, Santa Lucia Foundation, Rome, Italy

**Keywords:** IL-1 family cytokines, IL-1 family receptors, neuroinflammation, neurodegeneration, Alzheimer’s disease, multiple sclerosis

## Abstract

Cytokines and receptors of the IL-1 family are key mediators in innate immune and inflammatory reactions in physiological defensive conditions, but are also significantly involved in immune-mediated inflammatory diseases. Here, we will address the role of cytokines of the IL-1 superfamily and their receptors in neuroinflammatory and neurodegenerative diseases, in particular Multiple Sclerosis and Alzheimer’s disease. Notably, several members of the IL-1 family are present in the brain as tissue-specific splice variants. Attention will be devoted to understanding whether these molecules are involved in the disease onset or are effectors of the downstream degenerative events. We will focus on the balance between the inflammatory cytokines IL-1β and IL-18 and inhibitory cytokines and receptors, in view of future therapeutic approaches.

## 1 Introduction

The interleukin-1 (IL-1) superfamily encompasses eleven structurally and evolutionarily related cytokines that mainly act by binding to specific receptors (1). The IL-1 receptor (IL-1R) complexes are formed by a ligand-binding chain and an accessory chain necessary for signal transduction. The IL-1R family comprises ten related transmembrane molecules with extracellular Ig-like domains and intracellular TIR (Toll-Like Receptor/IL-1 Receptor) domains (2). Most IL-1 and IL-1R molecules are involved in inflammation/immunostimulation and its regulation. It is notable that, while IL-1R are derived from invertebrate TIR-containing receptors involved in innate immunity, IL-1 cytokines only appeared in vertebrates (likely linked to the development of adaptive immunity) and the majority of them is only present in mammals (3, 4).

In mammals, eleven cytokines of the IL-1 family have been identified, the inflammatory IL-1α, IL-1β, IL-18, IL-36α, IL-36β, IL-36γ, and the regulatory/anti-inflammatory IL-1Ra, IL-33, IL-36Ra, IL-37 and IL-38 (overview in 1). IL-1 family cytokines mainly act by binding to receptor complexes, composed by a ligand binding chain and an accessory signal transducing chain. Signalling is initiate by the pairing of the intracellular TIR domains of the two receptor chains. Receptors of the IL-1R family are ten structurally related transmembrane proteins, which may also give rise to soluble receptors upon gene splicing or proteolysis of the membrane chains (overview in 2). IL-1R1 binds the agonist ligands IL-1α and IL-1β, and its signalling depends on the engagement of the accessory chain IL-1R3. IL-1R1 also binds IL-1Ra but in this case fails to recruit the IL-1R3, and no activation occurs. Binding of IL-38 has also been reported. IL-1R2 is similar to IL-1R1 in its ligand binding capacity and IL-1R3 engagement, but it lacks the intracellular TIR domain and its therefore an inhibitory receptor. IL-1R4 is the receptor for IL-33, an uses the same IL-1R3 as accessory chain for signalling. IL-1R5 is the receptor for IL-18, and uses its specific accessory chain IL-1R7. It was reported that IL-37 also binds to IL-1R5 but is unable to recruit IL-1R7. IL-1R6 is the ligand binding chain for all the IL-36 isoforms (IL-36α, IL-36β, IL-36γ) and uses the promiscuous IL-1R3 as accessory chain. IL-1R6 also binds the receptor antagonist IL-36Ra and fails to engage the accessory chain. Binding of IL-38 has also been reported. IL-1R8 is an inhibitory receptor, which can interact with several IL-1R complexes and interfere with their signalling and may also act as inhibitory accessory chain for IL-37 bound to IL-1R5 and IL-38 bound to IL-1R6. IL-1R9 is an orphan receptor, although binding to IL-38 has been reported, predominantly expressed in neurons and likely involve in IL-1-independent neuronal functions. Likewise, IL-1R10 is abundantly expressed in the brain and has no IL-1-dependent functions (for complete references, please see the reference lists of 1, 2).

IL-1 and IL-1R molecules expressed in brain have as their main role the modulation of neuronal plasticity and function, in addition to mediating immune protective functions, in both physiologic and pathologic condition (5–8). IL-1, originally described as endogenous pyrogen or leukocytic pyrogen, is mainly produced in the hypothalamus in response to inflammatory agents and can induce fever by upregulating cyclooxygenase-2, the enzyme responsible for the synthesis of the vasoactive and inflammatory prostaglandin E_2_ (9, 10). A central nervous system (CNS)-restricted expression of splice variants of different IL-1 and IL-1R family members, as truncated forms with regulatory functions, have been identified for both IL-1 (11) and IL-18 (12–14). In the case of IL-37, only one of its five splice variants (IL-37a) is present in the brain (15). Some IL-1R members are only present in the brain, including a particular variant of the accessory receptor chain IL-1R3 (IL-1R3b), expressed in neurons and involved in IL-1-mediated neuroprotection. Notably, the receptor complex for IL-1α and β in the brain can be the canonical one (IL-1R1 plus IL-1R3, present everywhere in the body) that mediates inflammatory effects, or the alternative one (IL-1R1 plus IL-1R3b, specific for the brain) that mediates neuroprotective effects (2, 16–18). Other brain-specific receptors are the two orphan receptors IL-1R9 and IL-1R10, which are mostly expressed in neurons. IL-1R9 is likely involved in memory and learning capacity, as its mutations are linked to X-linked mental retardation and autism (2, 19, 20). The functions of IL-18 in the brain are also peculiar, since the cytokine is active (inflammation and homeostasis) even in the absence of its receptor IL-1R5, while IL-1R5 seems to be involved in the pathogenesis of experimental allergic encephalomyelitis (EAE, the mouse model of multiple sclerosis) in the absence of its IL-18 ligand (21, 22). This suggests that a CNS-specific network of IL-1 family cytokines and receptors is crucial in maintaining brain homeostasis, thus highlighting the limitation of a simplistic inflammatory paradigm to explain the function of these cytokines in neuroinflammatory diseases.

In this perspective, we examine the available experimental and clinical evidence of the involvement of these molecules in two major neurodegenerative diseases, Alzheimer’s disease (AD) and multiple sclerosis (MS), and propose a more global picture of the pathogenic and pathological role of the IL-1/IL-1R system.

## 2 Alzheimer’s disease

### 2.1 Disease pathogenesis and inflammation

Alzheimer’s disease (AD) in its sporadic form is the most common dementia diagnosis, characterized by a long progressive process of neuronal damage that affects memory and thinking and ultimately leads to death, in general preceded by a preclinical condition named mild cognitive impairment (MCI) (23).

The main pathological hallmarks of AD are the extracellular accumulation of amyloid β (Aβ) peptides and the intraneuronal neurofibrillary tangles (NFT) formed by aggregation of hyperphosphorylated tau proteins. Neuroinflammation is the third core feature of the disease (24, 25). Many inflammation-related genes have been identified as important AD risk factors (26), and increased inflammatory responses in the brain and periphery have been widely reported in AD (27, 28). Innate immune cells, primarily brain-resident microglia, have a dual role in AD neuroinflammation (29–33). Microglia have a protective function by releasing neurotrophic factors and clearing misfolded proteins, and can degrade Aβ thereby reducing its accumulation. Indeed, a transition of microglia from homeostatic to disease-associated populations endowed with protective potential has been described as a function of the disease progression (34). However, during the course of AD, microglia can be hyperactivated by accumulating danger-associated molecular patterns (DAMPs), mainly Aβ and tau, and secrete neurotoxic and inflammatory cytokines that cause persistent neuroinflammation and initiate/exacerbate neurodegeneration (35–38). The focus on neuroinflammation and inflammasome activation as cause of neuronal damage in AD (39–43) leads to assessing the role of the inflammatory cytokines generated by inflammasome activation, in particular the IL-1 family cytokines IL-1β and IL-18 (44).

#### 2.1.1 IL-1

In the brain, IL-1β is required for learning and memory processes, but, when expressed at aberrant levels, it is involved in infection- and sterile inflammation-induced cognitive dysfunction (45, 46). It has also been suggested that low levels of IL-1 act in the CNS to perform non-immunological functions, while higher concentrations could engage brain non-neuronal cells to produce neuroinflammation (47). Notably, brain IL-1β is produced by and target different cells, ranging from microglia, infiltrating leukocytes, astrocytes, to neurons. By activating different specific cell types, including endothelial cells, neurons and choroid plexus cells, IL-1 may exert different functions of both immunological and non-immunological nature, and neuroinflammation itself may be the result of multiple responses to IL-1 by different brain cell types (8).

In AD, IL-1β is an important mediator of neuroinflammation (48, 49), but also a protective factor (50), able to influence the balance between beneficial and detrimental outcomes (51). In mice lacking the gene encoding the IL-1 inhibitor IL-1Ra the AD-like pathology is exacerbated (52), while the IL-1R1 blockade results in decreased neuroinflammation, attenuated tau pathology and reversal of cognitive deficits (53). Modulation of levels of IL-1Ra or different IL-1R has been observed in brain and blood of AD patients (54–58). Overall, the available experimental and clinical data do not support an exclusively detrimental role of IL-1-driven inflammation in AD.

#### 2.1.2 IL-18

IL-18, its receptors IL-1R5 and IL-1R7 and its inhibitory protein (IL-18BP) are normally expressed in the brain and modulated in both experimental and clinical AD conditions (21). Similar to IL-1, IL-18 could exert both detrimental and protective functions in AD. Thus, while IL-18 dysregulation has been observed in AD patients in association with severity of symptoms and correlated to increased Aβ production *in vitro* (59–62), a protective function has been also reported in IL-18-deficient mice in both physiological (63) and AD-like conditions (64).

#### 2.1.3 IL-33

A third IL-1 family cytokine, IL-33, is being explored in CNS diseases (65). IL-33 is constitutively expressed in astrocytes and to a minor extent in oligodendrocytes, microglia and neurons; its receptor IL-1R4 is expressed on astrocytes, endothelial cells, glial cells and neurons (6). IL-33 can induce microglia cell proliferation and production of inflammatory IL-1β and TNFα, and anti-inflammatory IL-10 (66). In animal models, IL-33 decreases inflammatory responses and improves AD-like pathology (67). In patients, IL-33 expression in the brain is variably modulated relative to healthy subjects (68, 69). Patients with measurable circulating IL-33 levels have preserved cognitive functions, compared with those that do not express the cytokine (70). Eventually, in MCI patients treated with the neuroprotective compound homotaurine, the improvement of cognitive functions correlates with increased IL-33 plasma levels (71), confirming the potential protective role of this cytokine in AD development.

### 2.2 Mechanisms of IL-1/IL-1R family in AD pathology

IL-1β, IL-18 and IL-33 are dysregulated in AD and likely exerting a dual role: driving the inflammatory pathogenic processes associated with the disease and providing protection to the damaged CNS. The functions of these mediators in brain homeostasis and pathology can be pleiotropic, redundant, synergic and cross-regulating, aiming to switch off or amplify the inflammatory response in a context-dependent manner. In this scenario, the IL-1R signaling could lead to the production of inflammatory cytokines and participate in Aβ-induced inflammasome activation in microglia, while it might even increase the Amyloid Precursor Protein non-amyloidogenic processing in neurons, thereby preventing neurotoxic Aβ generation (72). Overall, whether and when the different molecules belonging to the IL-1/IL-1R family are either beneficial or detrimental to neuronal function in the course of AD, and how their negative feedback mechanisms influence this, is still a matter of intense research (39). However, the general deregulation of IL-1 family members observed in patients consistently reflect the AD-linked activation of the innate immune system, strongly suggesting that an inflammatory condition, although not exclusively detrimental or beneficial, is indisputably present in AD.

It is likely that neuroinflammation is both a response to Aβ and tau NFT that exacerbates their deleterious effects, and a cause of the disease, that increases brain deposit of Aβ and tau phosphorylation starting before onset of symptoms (73, 74). Convincing new data in patients show that microglia activation and consequent neuroinflammation are associated with disease development and progression (75). The infectious hypothesis (76, 77) offers a fascinating explanation of the etiological meaning of inflammation in the disease and the underlying role of IL-1 family cytokines. According to the observation that Aβ has antimicrobial functions (78), several microbial infections (herpesviruses, periodontal bacteria, gut microbiota dysbiosis) have been associated to AD. By inducing inflammasome activation, infectious agents can induce the release of IL-1 family cytokines, which can contribute to blood brain barrier alterations, amplify pre-existing inflammation in the brain, influence Aβ pathology (note that infections upregulate Aβ production) and tau-related neurodegeneration. Indeed, it has been proposed that the AD pathology can be modulated by innate immune mechanisms associated with infectious burden, including long-term innate memory, which affect epigenetic reprogramming of microglia and the release of IL-1 family cytokines (79, 80).

## 3 Multiple sclerosis

### 3.1 Pathology and inflammation

Multiple sclerosis (MS) is a chronic inflammatory neurological disorder driven by myelin-targeting CD4^+^ T cells. The two main disease phenotypes are the relapsing-remitting form (RRMS) and the primary progressive MS, with a steady loss of function (PPMS) (81, 82). RRMS patients may evolve to a progressive disease course, diagnosed as secondary progressive MS (SPMS). The characteristic demyelinating lesions are localized in both white and grey matter, and contain an abundant inflammatory infiltrate with activated macrophages, microglia, CD8^+^ and CD4^+^ T cells, B and plasma cells (83). Inflammatory mediators released by innate and adaptive immune cells lead to damage of oligodendrocytes, the cells responsible for myelin deposition, and axonal injury. A wealth of information, in particular in experimental models, suggests a role for the gut microbiota in bidirectionally modulating brain inflammation in MS, with changes in bacterial composition inducing upregulation *vs*. downregulation of the production of IL-1β and other inflammation-related factors (84–87).

#### 3.1.1 IL-1β

Acting on astrocytes and endothelial cells, IL-1β increases the permeability of the blood-brain barrier and allows leukocyte recruitment (88); acting on T cells, it induces the encephalitogenic phenotype of Th17 (89). *IL1B* transcript and IL-1β protein are detected in CNS lesions of MS patients (88), and microglia display an intense IL-1β staining (90). Changes in IL-1 levels in sera and cerebro-spinal fluid (CSF) are non-univocal, possibly due to patients’ heterogeneity and the interference of therapies. Elevated serum levels of IL-1β were reported (91), and the difference with normal subjects was higher for untreated RRMS patients with active disease (92). Other authors report normal levels of IL-1α and IL-1β in active patients, who however had received immunosuppressive therapy (93). Conflicting data have been obtained also in the analysis of CSF. Markedly increased levels of IL-1β in CSF were reported by some authors (91, 94) but not confirmed by others (95, 96). The possible relationship between IL-1β levels and disease activity is also challenged by the observation that in a group of active patients neither serum nor CSF levels were affected by high dose steroid treatment (91).

Soluble inhibitors and decoy receptors are also modulated in MS. IL-1Ra, undetectable in normal CNS, is expressed in lesional foamy macrophages, which also express the anti-inflammatory factors IL-10 and Transforming Growth Factor β, and markers typical of anti-inflammatory M2 macrophages (97). IL-1Ra levels are significantly higher in CSF but not in serum, and even if not correlated with disease activity, they are further increased by steroid treatment (91). IL-1Ra levels are strongly related with disability index, suggesting the potential role of IL-1Ra as serum biomarker of disease progression (98). On the contrary, the soluble decoy receptor IL-1R2 is not elevated in serum compared to normal controls, but steroid treatment induces an increase of its levels (91). In CSF, sIL-1R2 is undetectable irrespective of the disease stage. Finally, soluble IL-1R3 levels are significantly elevated in sera and CSF of MS patients, but not related to disease activity and not modulated by therapy.

#### 3.1.2 IL-18

IL-18 levels were elevated in sera (99–101) and also in CSF, especially in patients with active lesions (99). The findings in CSF, however, were not confirmed in other studies (102). An increase in serum IL-18 levels is detected in untreated RRMS (93) but also in SPMS (102). As observed in most inflammatory disorders, the increase in IL-18 serum levels is accompanied by a parallel increase in its soluble inhibitor IL-18BP, aimed at counteracting the IL-18 inflammatory effects. Although free IL-18 was not evaluated, the IL-18BP/IL-18 ratio was higher in RRMS patients than in controls (93).

#### 3.1.3 Other IL-1 family members

IL-33 has been extensively investigated in MS and EAE. The expression levels of both the cytokine and its receptor IL-1R4 are increased in lesions of MS patients and EAE mice (65, 103). Serum levels of IL-33 are elevated in patients and decreased by treatment with IFNβ (103). Longitudinal assessment of peripheral expression of IL-33 in RRMS revealed peak expression after relapses, but a clear correlation with clinical recovery was not demonstrated (104). In EAE mice, administration of IL-33 attenuates the disease, while antibody-mediated or genetic blockade exacerbates it (105–107). Similarly, IL-1R4 deficient mice develop a severe form of EAE. In this model, IL-33 induces M2 macrophage polarization indirectly, by stimulating mast cells to produce IL-13, and M2 cells then limit the expansion of pathogenic Th17 cells (108). The contribution of IL-1R4^+^ Treg, activated by IL-33, should also be taken into account. Controversial results have been obtained on the direct effect of IL-33 on myelination (109, 110), but on the whole the data are consistent with the notion that IL-33 is involved in neuroprotection and repair.

Interesting data underline the role of IL-37, an anti-inflammatory member of the IL-1 family. Intracellularly, IL-37 acts as an anti-inflammatory transcriptional regulator. Extracellularly, IL-37 signaling is allegedly mediated by a receptor complex formed by IL-1R5 and IL-1R8. IL-37 is barely detectable in PBMC from normal or MS patients; in the brain, its low expression levels are further downregulated in active MS lesions (111). Notably, the level of IL-37 gene expression in PBMC of MS patients is associated with a lower number of relapses (112). IL-1R5 and IL-1R8 are expressed at similar levels in PBMC and brain from normal or MS patients, and the levels of IL-1R5 (which is also the receptor for IL-18) tend to increase in active lesions. In sera, IL-37 is detectable in 8% of RRMS patients in a stable disease phase and 42% in the active stage; its levels are increased by treatment with a disease-modifying agent, fingolimod (112). Data from patients suggest that the anti-inflammatory activity of IL-37 may be defective in MS, as also confirmed by animal experiments. EAE is milder in transgenic mice expressing human IL-37, with reduced inflammatory infiltrate and decreased numbers of Th1 cells and inflammatory macrophages in lesions (111). This beneficial effect of IL-37 on EAE is lost in mice lacking IL-1R8, indicating that the cytokine confers protection acting through the inhibitory receptor IL-1R8. Moreover, administration of recombinant IL-37 reduces neurological deficits and demyelination in EAE mice. Thus, IL-37 is protective in neuroinflammation and may represent a novel treatment in MS.

## 4 The NLRP3 inflammasome

The NOD-like receptor 3 (NLRP3) inflammasome, a multiprotein complex that regulates production and release of inflammatory cytokines, in the brain is mainly expressed in microglia but also in astrocytes and neurons. Besides being involved in protection from bacterial, fungal and viral agents, NLRP3 plays a role in neurodevelopment and neuroprotection, but its aberrant activation is crucial in neuroinflammation (113).

Different pathways, including impaired autophagy and dysregulated cell death mechanisms, upregulate NLRP3 activity in AD (114). Aβ and tau can directly activate NLRP3 in microglia (115–117), and several compounds that ameliorate AD-associated pathology reduce NLRP3 activity.

Strong evidence points to a critical role of inflammasome activation in MS. Caspase-1 is more expressed in peripheral blood mononuclear cells (PBMC) of MS patients than in controls (118). Its serum levels can be considered a biomarker of MS, even if they do not identify more active or more severe patients (101). In untreated patients, upregulation of NLRP3 gene expression and higher caspase 1 activity were detected in circulating monocytes (119). NLRP3 upregulation characterized patients with PPMS and was associated with hyperexpression of several inflammatory chemokines and cytokines. IL-1β was one of the most expressed genes, correlated with faster disease progression in PPMS (119). There is a marked increase in the number of IL-1β, caspase-1, and gasdermin D (GSDMD)-positive cells in white matter of MS versus non-MS patients. GSDMD, activated by inflammatory caspases, forms membrane pores responsible for pyroptosis an inflammasome-driven programmed cell death (120). In MS lesions, inflammasome activation and pyroptosis are detected in microglia and oligodendrocytes, suggesting a role in demyelination. Inhibition of inflammasome activation and caspase-1-dependent IL-1β production have already been exploited in MS therapy: IFNβ treatment reduces NLRP3 activity and induces IL-10, thus decreasing the production of pro-IL-1α, pro-IL-1β and mature cytokines (121).

In experimental autoimmune encephalomyelitis (EAE), the mouse MS model, blockade of caspase-1 expression or activity attenuates neuroinflammation (122). Caspase-1 inhibition also reduces GSDMD activation and pyroptosis, limiting oligodendrocyte death and demyelination (120). Similarly, NLRP3 KO or IL-18 KO mice have a less severe disease course, with a marked reduction of CNS inflammatory infiltrate (123, 124). Microglia-specific deletion of the gene encoding the NFκB regulatory protein A20 leads to microglial cell proliferation, susceptibility to LPS-induced inflammation and increased EAE severity, reversed by NLRP3 or caspase-1 genetic deletion (125), indicating a critical role of microglia in MS, mediated by inflammasome activation. The inflammasome involvement in neuroinflammation is also suggested by data in a cohort of MS patients, in which low penetrance mutations of the NLRP3 gene were detected in 16% of the cases (126). These mutations are associated with mild symptoms (suggestive of autoinflammatory disease) and increased production of IL-1β and IL-18 (127).

## 5 IL-1 family molecules in other main neurodegenerative diseases

Inflammation is involved in other neurological diseases, both neurodegenerative (see below) and of traumatic or other origin (trauma, stroke, autism, schizophrenia, epilepsy). Focusing on neurodegenerative disorders, these are classified in four main groups: amyloidoses, tauopathies, alpha-synucleinopathies, and TDP (transactivation response DNA binding protein)-43 proteinopathies, all characterised by abnormal protein conformations and the formation of protein aggregates in neuronal cells in different brain areas (128, 129). AD is the most common and prevalent of both amyloidoses and tauopathies, along with frontotemporal dementia with parkinsonism and some forms of prion diseases. Main α-synuclein-related diseases include Parkinson’s disease (PD), Dementia with Lewy Bodies, and Multiple System Atrophy, while Amyotrophic Lateral Sclerosis (ALS) is mainly associated with TDP-43 pathology. The Huntington disease (HD) is associated with mutations in the huntingtin protein, which clumps within neurons provoking cell damage and death (130).

Although it is well known and documented that all neurodegenerative diseases share the chronic aberrant inflammation (131), less is known on the role of IL-1 family cytokines and receptors in specific neurodegenerative diseases (132). In addition to AD and MS, reviewed in the previous sections, here we will briefly examine the involvement of IL-1 family molecules in PD, HD, and ALS.

PD is characterised by the death of dopaminergic neurons in the *substantia nigra* and by the cytoplasmatic aggregation of fibrillar α-synuclein in Lewy bodies (133). Increase of α-synuclein released outside the cells drives the activation of microglia, and the microglia-dependent production of TNF-α, NO, and IL-1β sustains the neuroinflammatory process in PD (134). Recent results in the 6-OHDA mouse model showed that the increased levels of IL-1β and TNF-α have a different kinetics, with TNF-α appearing in the advanced disease stage, while increased level of IL-1β in serum were already evident with a moderate degree of lesion in *substantia nigra*, suggesting a prognostic value for this inflammatory cytokine (135). Although the results on the level and role of IL-1β in PD patients are conflicting (136), the activation of the NLRP3 inflammasome seems to be determinant for the development of PD at least *in vitro*. Indeed, when activated, the inflammasome can cause the aggregation of α-synuclein in a neuronal cell model of PD, as caspase-1 directly cleaves α-synuclein generating a truncated protein highly prone to aggregation and able to provoke cell death (137). The involvement of the NLRP3 inflammasome activation in PD also includes two additional mechanisms, the interaction with the parkin protein, whose mutations are responsible of autosomal recessive familial and sporadic early-onset PD (138, 139); and the association with mitochondrial dysfunction that affects neuron performance and favours neuronal degeneration (140). Although IL-1β is a major product of the NLRP3 inflammasome activation, its role in causing/sustaining inflammation in PD patients is still debated (141).

Increasing evidence suggest that inflammation contributes to the development of HD, but the involvement of IL-1 family is still poorly known. The role of IL-1β has been investigated in mice expressing a mutant and pathogenic huntingtin crossed with animals knocked out for IL-1R1 (142). These mice showed more severe neurological symptoms compared to control mice expressing IL-1R1, suggesting a protective role for IL-1R1 signalling in preventing the HD neuropathology. Whether IL-1β is the molecule that activates IL-1R1 signalling is not known. Although IL-1β has been identified as one of the inflammation-related markers of the disease in the porcine HD model (143), its level does not increase in plasma of HD patients, at variance with IL-6 or other inflammatory factors (144, 145). An involvement of the NLRP3 inflammasome has been also suggested for HD, although evidence is still poor and mostly from pre-clinical studies (146).

ALS is a neuromuscular disorder characterized by the progressive loss of anterior-lateral horn spinal cord motoneurons (147). Sporadic ALS affects the 90-95% of patients, while the familiar form affects the remaining 5-10% of patients. The two most relevant genetic mutations associated with ALS are a defect in the hexanucleotide repeat expansion (HRE) in intron 1 of the *C9orf72* gene (which makes a protein that regulates actin dynamics and endosomal recycling of GluR1 at the synapse) (148), and in the free radical scavenging enzyme Cu,Zn-superoxide dismutase (SOD1) gene (149). The involvement of inflammation in the etiopathogenesis of ALS is starting to be investigated, but studies are still limited (150, 151). In a recent study, the authors suggest hyperinflammation and immunodeficiency as primary triggers of motoneuron death in ALS, and underline the complex link between the development ALS and T cell and monocyte profiles of patients, as well as polymorphisms in cytokine and chemokine receptors, suggesting a need for personalised therapies based on immunophenotyping (152). In this regard, recently new polymorphisms in the gene encoding IL-1β and in genes involved in oxidative stress (*e.g.*, SOD2) have been identified as modifiers of ALS progression (153). IL-1β is one of the main biomarkers of inflammation in ALS (151, 152). Even if it is not the best marker of ALS severity (154), its plasma levels negatively correlate with survival in ALS patients with genetic variant *C9orf72HRE* (155). Among the IL-1 family cytokines and receptors, it has been observed that only IL-18 is associated with sporadic ALS, whereas the serum levels of the cytokines IL-33 and IL-36 and the soluble receptors sIL-1R2 and sIL-1R4 were comparable between ALS patients and healthy controls, and IL-1β, IL-1Ra and IL-37 were below detection (156). The possible pathogenic role of IL-18 in ASL has been also recently underlined by a Whole Genome Sequencing study, which identified a genetic variant in a noncoding region of the gene encoding the IL-18 receptor accessory protein (*IL18RAP*) able to reduce mRNA stability and motor neuron neurotoxicity, thereby decreasing 5x the risk of developing ALS (157). A protective role has been suggested for another member of IL-1 family, IL-33 (158). In the ALS mouse model transgenic for G93A-superoxide dismutase 1 (*SOD1-G93A*), long-term IL-33 administration delays disease onset in females but not males, probably through peripheral Th2 response (159).

## 6 Conclusions and perspectives

The role of IL-1 family cytokines and receptors in the etiology and pathogenesis of neurodegenerative diseases is still poorly understood. However, there is strong evidence of their involvement in the initiation and regulation of inflammation associated to most CNS diseases, and their participation to neuroinflammation has been widely described in AD an MS. This is evident despite the limitations due to the current lack of reliable animal models that realistically recapitulate the pathological features of the human diseases, such as in the case of sporadic AD (160). In humans, longitudinal molecular monitoring of inflammation is also hampered by the reduced accessibility of human brain and the limited availability of clinical studies with extensive follow-ups (161, 162).

From the combined *in vivo* and *in vitro* functional and molecular evidence, it is clear that these factors play a homeostatic and neuroprotective role in healthy conditions. It is notable that the IL-1 family system in the CNS includes several molecules and functions that are CNS-restricted, implying tissue-specific needs and consequent functional adaptation. Disease conditions imply an imbalance of the IL-1 system network (**Table 1**).

**Table 1:** IL-1 family cytokines and receptors in brain physiology and pathology.

Cytokine/ receptor	Physiological role	Role in pathogenesis	Role in pathology	References
IL-1β	• Learning and memory processes •Thermoregulation	• Induction of inflammation •Induction of fever	• Persistence of inflammation •Neurodegeneration	(7–10, 45–51, 88–92)
IL-1Ra	• Unknown (regulation of IL-1 activity)	• Unknown	•Increased with disease (feedback mechanism)?	(52, 91, 97, 98)
IL-18	•Neuroprotective	•Induction of inflammation	•Persistence of inflammation	(21, 59–64)
IL-18BP	• Unknown (regulation of IL-18 activity)	• Unknown	• Increases with disease (feedback mechanism)?	(93)
IL-33	• Regulates microglia proliferation • Regulates the microglial inflammatory/anti-inflammatory balance	• Anti-inflammatory protective effects	• Upregulated in pathology • Negative correlation with disease severity	(6, 65–71, 103, 108)
IL-37	• Unknown	• Anti-inflammatory protective effects	• Downregulated in active disease • Negative correlation with disease severity	(111, 112)
IL-1R3b	• Accessory receptor mediating the neuroprotective effects of IL-1	• Unknown	• Unknown	(16, 18)
IL-1R4	• Mediates the homeostatic activities of IL-33	• Unknown	• Soluble form upregulated during disease (feedback mechanism)?	(6, 108)
IL-1R5	• Mediates the homeostatic activities of IL-18	• Associated with disease pathogenesis in EAE	• Unknown	(21, 111)
IL-1R8	• Anti-inflammatory orphan receptor; physiological role unknown	• Unknown	• Necessary for the neuroprotective role of IL-37	(111)
IL-1R9	• Orphan receptor, involved in learning and memory processes	• Unknown (mutations are associated with X-linked mental retardation, schizophrenia and autism)	• Unknown	(19, 20)

From the available experimental and clinical evidence, we can draw the following conclusions and suggestions.

**Figure 1:**
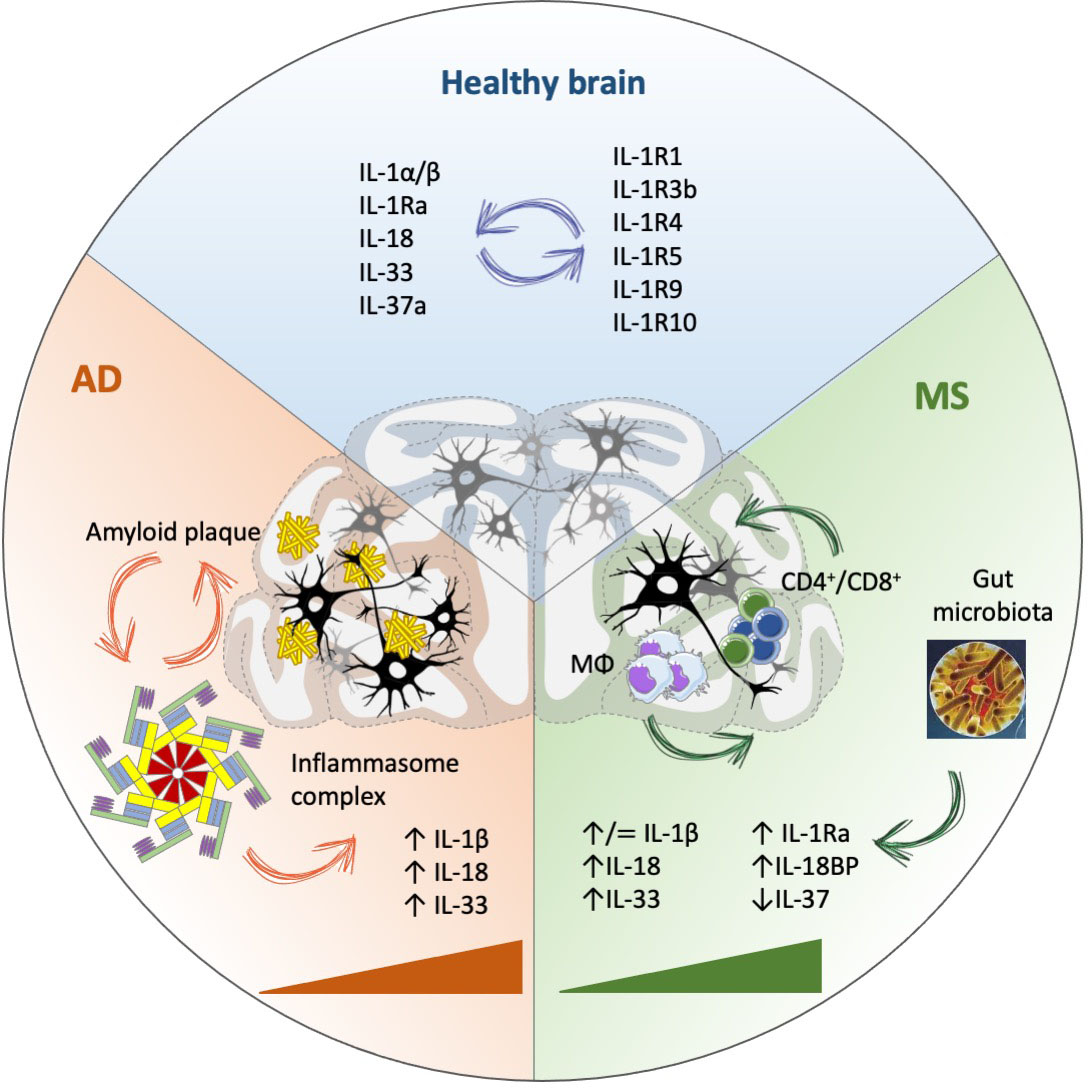
IL-1 family involvement in Alzheimer’s disease (AD) and Multiple Sclerosis (MS). IL-1 family cytokines and receptors are crucial in maintaining brain homeostasis, with an important role in the modulation of neuronal plasticity and function, in addition to mediating immune protective functions. In physiological conditions, many activities of IL-1 family cytokines depend on binding to specific receptors, but independent functions have been observed for both cytokines (*e.g.*, IL-18) and receptors (*e.g.*, IL-1R9). In disease conditions (AD, MS), IL-1 family members are substantially modulated during disease progression (triangles), associated with increased inflammation (partly dependent on inflammasome activation) and likely involved in all disease phases, from initiation and establishment to progression, without a clear association with either disease initiation or progression. MΦ, macrophages.

• IL-1 family cytokines and receptors are altered in AD and MS and related to the neuroinflammatory conditions.
• Alterations in the amount of produced inflammatory cytokines (IL-1, IL-18) likely switch their role from neuroprotection/homeostasis to pathological inflammation.
• The excess of inflammatory cytokines causes an excessive induction of feedback mechanisms, *e.g.*, the production of downregulating factors (IL-1Ra, IL-18BP, IL-33, IL-37), which however do not succeed in rebalancing the excessive inflammation.
• Unbalanced IL-1 family molecules contribute to alterations in adaptive immune responses, thereby amplifying the autoimmune aspects of neurodegenerative diseases.
• Whether anomalies in the IL-1 family cytokine and receptor network are cause or effect of neuroinflammation and neurodegeneration in AD and MS is not an appropriate question, as it seems that these factors are involved in all steps of disease, from its induction to its establishment and downstream symptoms (**Figure 1**).
• The CNS-restricted peculiarities of the network of IL-1 family cytokines and receptors suggest a tissue-specific physiological balance and pathological dysregulation. A more thorough understanding of the CNS specificities in the IL-1 family system will open the way to a precision rebalancing approach for therapeutic purposes.

## Author contributions

DB, PM, PI and PB wrote the manuscript and critically revised it. DB and PI contributed to figures and tables. All authors contributed to the article and approved the submitted version.
